# A preliminary exploration on DNA methylation of transgene across generations in transgenic rats

**DOI:** 10.1038/srep08292

**Published:** 2015-02-06

**Authors:** Qiling Li, Wei Xu, Ye Cui, Li Ma, Jendai Richards, Wenzhi Li, Yamin Ma, Guoxing Fu, Tameka Bythwood, Yueling Wang, Xu Li, Qing Song

**Affiliations:** 1 Department of Obstetrics and Gynecology, First Affiliated Hospital of Xi'an Jiaotong University, Xi'an, Shaanxi, China; 2 Cardiovascular Research Institute, Morehouse School of Medicine, Atlanta, Georgia, USA; 3 Department of Mathematics and Statistics, Georgia State University, Atlanta, Georgia, USA

## Abstract

Epigenetic heritability is an important issue in the field of genetics and also in the development of many human diseases. In this study, we created a transgenic rat model and investigated the transgenerational methylation patterns in these animals. The transgene DNA fragment was unmethylated before it was injected into the pronucleus, so it is a good model to study the inheritance of DNA methylation patterns. We performed bisulfite sequencing on 23 CpG dinucleotides on the transgene across three generations in two tissues. We observed that the transgene was heavily methylated in the liver (87.53%) from the founder generation, whereas its methylation rate was much lower in the kidney (70.47%). Spearman correlation analysis showed that there was a strong correlation on the methylation status between different generations in the same tissue, which was observed in both liver and kidney, and among all individuals in this pedigree. This study provided some evidence that DNA methylation patterns acquired in the founder animal can be passed to the offspring.

Since the discovery of the double-helix structure 60 years ago, it has been known that genetic information can be passed from generation to generation using DNA molecules as the carrier. However, accumulating evidence has showed that some acquired phenotypic traits from environmental stresses can also be inherited from parents to offspring across the generations^1^,^2^,^3^. For example, it has been reported that specific acquired alterations of gene expression can be transmitted through the germline to most of the offspring^4^, and may confer a high risk of developing a disease^5^.

The cytosine of mammalian DNA can be chemically modified to methylated cytosine or hydroxymethylated cytosine. These chemical modifications can determine the three-dimensional conformation of chromosomal DNA, and thus the accessibility of DNA to transcription factors and other DNA-binding proteins, which is critical in the regulation of gene expression. There are many key questions yet to be answered regarding DNA methylation, especially about the transgenerational epigenetic heritability^6^. Transgenic animals provide us an opportunity to gain insight on the issue. In the process of generating the transgenic animals, the DNA fragment was released from a plasmid in which the cytosines are unmethylated. After these unmethylated DNA molecules were microinjected into the pronucleus, they are incorporated into the host genome and subjected to changes on their methylation status. In this study, we monitored the DNA methylation status in our transgenic rat model across generations.

## Results

### Validation of bisulfite conversion rate

We carried out bisulfite DNA sequencing specifically targeted at the nucleotide sequence of the human CRP transgene that was integrated into the rat genome. This region contains 31 CpG dinucleotides, the common targets for cytosine methylation. We successfully measured the methylation status of 23 CpG dinucleotides among the CpGs in this study. First, we determined the conversion rate in our bisulfite treatment experiments, which is regarded as the control experiment to confirm if the chemical conversion is successful. Because those cytosines that are not on the CpG dinucleotides are rarely methylated, by comparing the sequence results between the DNA templates before and after the bisulfite treatment on those non-CpG cytosines, we can measure the bisulfite conversion rate. In our experiments, we observed that about 99.4% of cytosine residues were successfully converted to thymidine, which confirmed that our bisulfite sequencing can reliably read out the methylation status on those CpG dinucleotides. Previous studies have shown the presence of non-CpG methylation phenomenon^7^,^8^; DNA methylation at cytosines in the context of CpA, CpT and CpC have been reported in embryonic stem cells, sensory neurons^9^ and plants^7^. Those non-CpG methylated cytosines (~0.6% observed in our result) may either represent true methylation on those CpA, CpT or CpG dinucleotides, or leaking in the bisulfite reactions. Nevertheless, this conversion rate is high and sufficient for the following methylation analysis.

### Differential methylation in the liver and kidney

**Figure 1:**
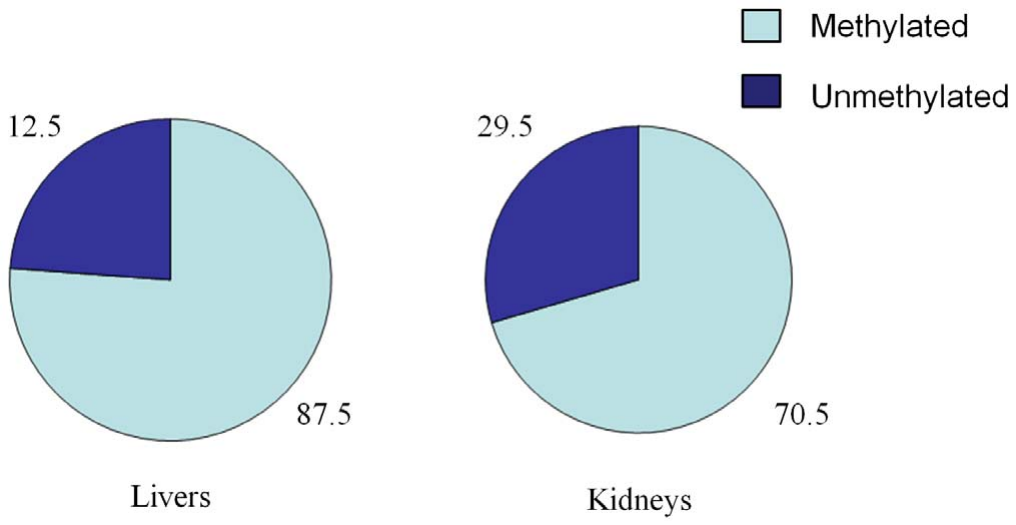
Overall methylation rates of transgene. About 87.53% CpG dinucleotides were methylated in the liver, and 70.47% in the kidney, there is a statistically significant difference between these two tissues (*P* < 0.001).

**Figure 2:**
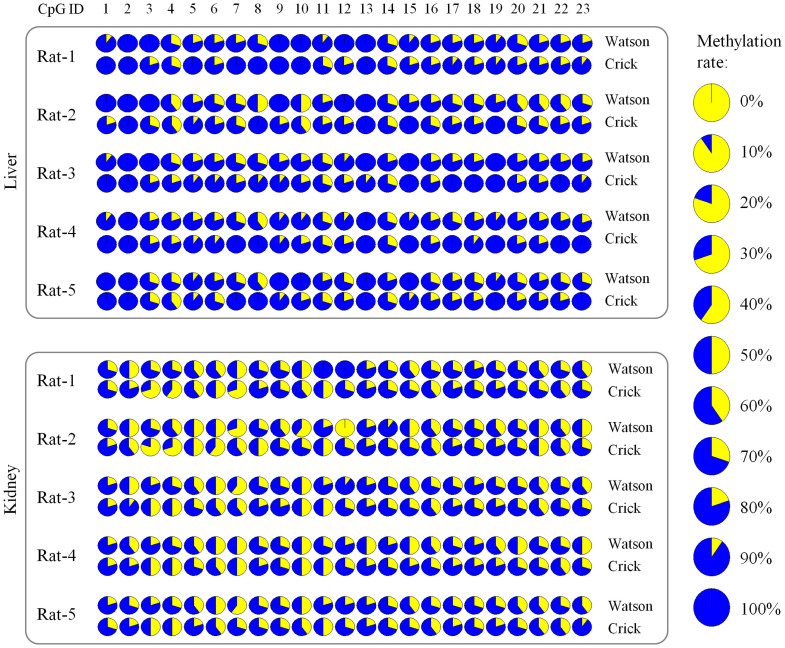
Quantitation of methylation status of transgene in the liver and kidney. Totally 23 CpG dinucleotides of 5 rats in three generations were studied in these two tissues. Each circle represents the methylation status on one CpG site of one DNA strand. The rate of DNA methylation of each CpG on each strand was quantitated from the peaks of Sanger bisulfite sequencing. The data of methylation status were shown on two strands (Watson and Crick) separately. Totally about 250 ng of genomic DNA was input into each experiment, which was equivalent to about 38,000 pooled DNA molecules. The average percentage of methylation on each CpG dinucleotide was measured by Sanger sequencing. In each circle, the blue color represents the percentage of unmethylated CpG at this site; the yellow color represents the percentage of methylated CpG at this site.

We measured the methylation status of 23 CpG dinucleotides on the transgene in the liver and the kidney. Among these 5 rats, we observed that the overall methylation rates were quite different between the liver and kidney (Figure 1, and Figure 2). In the liver, the transgene was methylated at 87.5% of CpG dinucleotides, but in the kidney, the transgene was methylated at a substantially lower level (70.5%). No significant difference was found between Watson and Crick strands among 115 CpG sites.

### Transgenerational similarity on the methylation status

We systematically analyzed the correlation of methylation status of the transgene in different generations. The overall methylation rates of three generations were not significantly different between three generations in both liver and kidney (Figure 2). We found that even though the original transgene was “naked” without any methylation before it was injected into the fertilized eggs, it was remarkably methylated in vivo in the founder rat and the offspring. To determine the correlation of methylation status at the same CpG sites on the transgene across generations, we performed the Pearson's correlation analysis. In the liver, we observed high correlations on all CpG dinucleotides, except in Rat-2 vs. Rat-4 in the kidney, and between different rats of different generations on the same CpG dinucleotides on both of the Watson strand and the Crick strand (Table 1). Although the overall methylation rates in the kidney were quite different from the overall rate in the liver (Figure 2), we observed that the correlation on CpG methylation status between different generations of rats was also strong in the kidney (Table 2). Our results indicated a high similarity of methylation status between different generations.

## Discussion

In this study, we examined the correlation of methylation status across three generations. The transgene DNA fragment in vitro had no DNA methylation before it was injected into the pronucleus of the fertilized eggs; thus, monitoring the methylation status of the transgene gave us an opportunity to investigate the inheritance of DNA methylation in different generations. We observed that after the transgene DNA was integrated into the host genome, it started to be heavily methylated since the founder generation. The transgene showed quite different methylation rates between the liver (87.53%) and the kidneys (70.47%). The correlation of the CpG methylation status between different generations is very strong in both the liver and the kidney. This study provided some data for the research community to investigate transgenerational heritability of DNA methylation.

Recent studies have suggested the heritability of acquired phenotypes. For example, it was reported that paternal high-fat diet consumption could induce common changes in the transcriptomes of retroperitoneal adipose and pancreatic islet tissues in female rat offspring^10^. In another example, an epigenetic event associated with the heterochromatic disruption induced by heat shock or osmotic stress was transmitted to the next generation in a non-Mendelian fashion^11^. When embryos were exposed to heat stress over multiple generations, the defective chromatin state was maintained over multiple generations and gradually returned to the normal state^11^. It has been proposed that DNA methylation may subsidize the heritability of these acquired traits.

Full methylation rates were also significantly different between the liver and the kidney (*P* < 0.001). It has been shown in plants that CpG methylation can be maintained not only in promoters but also in the body of transgene^12^, this is consistent with our results observed in animals. In our study, all of the 23 CpGs were located in both promoter and gene body regions of the transgene; our results showed that the methylation status of CpGs in the gene body can be maintained across generations. It has been estimated that the failure of maintenance was estimated to occur at a frequency of ~5% per CpG site per cell division^13^. In summary, this study provided some evidence on the heritability of DNA methylation across generations.

## Methods

### Creation of human CRP transgenic rats

**Figure 3:**
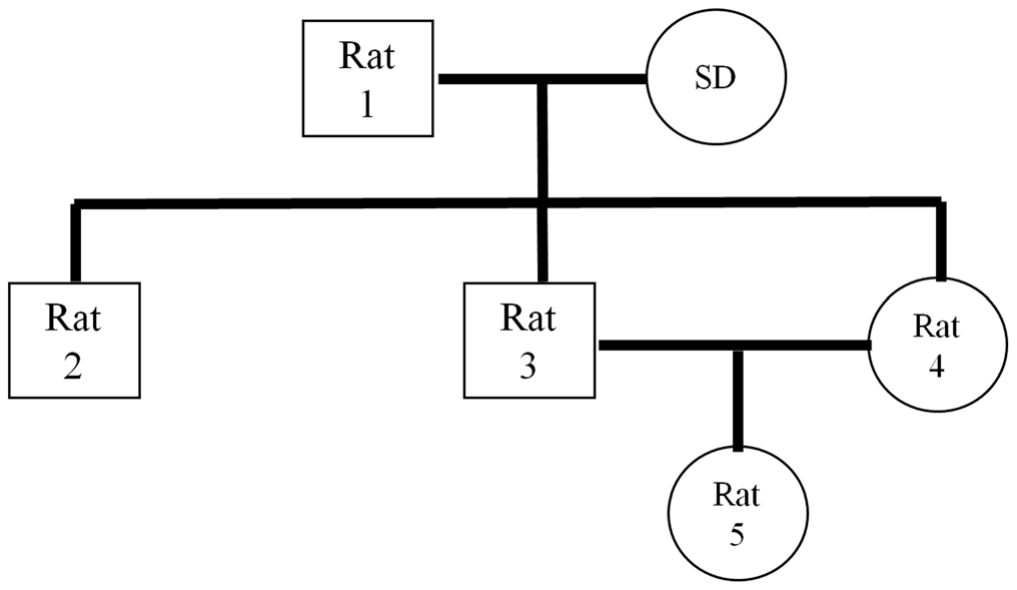
The three-generation pedigree of rats in this study. SD, Sprague-Dawley. Squares indicate male rats; circles indicate female rats. Rat ID was labeled in the squares or circles.

The transgene contained human *CRP* gene (21 bp fragment before the transcription starting site, the exons and intron, and 1.2 kb of 3′-flanking region), and mouse albumin promoter (from +22 to −305 bp) and enhancer (from −12.171 kb to −9.469 kb)^14^. Purified DNA was microinjected into fertilized eggs of Sprague-Dawley (SD) rats (Charles River Laboratory, Wilmington, MA). Pronuclear microinjection was performed at the University of Michigan Transgenic Animal Model Core Facility. Transgenic rats were identified by PCR with transgene-specific primers (Forward 5′-ACATACGCAAGGGATTTAGTC-3′; Reverse 5′-AACAGCTTCTCCATGGTCAC-3′) using genomic DNA samples obtained from tail biopsies. Founder rats were bred with non-transgenic SD rats to establish transgenic lines. Animals were housed in the Center for Laboratory Animal Resources of Morehouse School of Medicine. Transgenic *CRP* rats were given water ad libitum and a standard rat chow diet (Laboratory Rodent Diet 5001, LabDiet, USA). All animal experiments were performed with the approval of the Animal Care Committee of Morehouse School of Medicine, and conformed to the Guide for the Care and Use of Laboratory Animals published by the National Institutes of Health. Five transgenic rats at 15–16 weeks old in three generations were chosen in this study (Figure 3). Among these 5 rats, Rat-1 was a founder rat, Rats 2–4 were the second generation of heterozygous offspring, and Rat-5 was the third generation heterozygous offspring.

### DNA extraction

After the transgenic rats were anesthetized with 70% CO_2_, liver and kidney were collected. Genomic DNA was extracted using DNeasy blood& tissue kit following the manufacture's protocol (Qiagen). DNA concentration was measured using the Nanodrop spectrophotometer (Thermo Scientific, DE, USA).

### Genomic DNA bisulfite conversion

Bisulfite conversion is now the "gold standard" for assessment of DNA methylation status. Bisulfite treatment can deaminate specifically those unmethylated cytosine and convert them into uracil in DNA, while those methylated cytosines are protected by the methyl group in this chemical reaction from being converted into uracil. By comparing sequencing results of the original DNA molecules before the bisulfite treatment and the sequencing results after bisulfite treatment, the locations of unmethylated cytosines and 5-methylcytosines can be specifically determined at single-nucleotide resolution. In these experiments, genomic DNA (250 ng) was bisulfite-converted using EZ DNA Methylation-Direct Kit following the manufacture's protocol (Zymo Research). About 20 μl of sample was added to 130 μl of CT Conversion Reagent solution in a PCR tube, and then incubated in a thermal cycler with the cycling protocol: 98°C for 8 minutes and 64°C for 7 hours. Then the samples were processed with a series of buffers and the Zymo-SpinIC Column following the manufacturer's protocol.

### Purification of bisulfite converted DNA

Purification of PCR products was completed using QIAquick PCR purification kit (QIAGEN, 28106). Five volumes of Buffer PB was added to 1 volume of the PCR sample and mixed. A QIAquick spin column was placed into a 2-ml collection tube. To bind DNA, the sample was applied to the QIAquick column and centrifuged for 30–60 seconds. The flow-through was discarded, and the QIAquick column was placed back into the same tube. To wash, 0.75 ml of Buffer PE was added to the QIAquick column and centrifuged 30–60 seconds. The flow-through was discarded and the QIAquick column was placed back in the same tube. The QIAquick column was placed in a clean 1.5 ml microcentrifuge tube. To elute DNA, 50 μl of H_2_O was added to the center of the QIAquick membrane and the column was centrifuged at maximum speed for 1 minute.

### Amplification of bisulfite converted DNA

After bisulfite conversion, two complementary DNA strands are no longer complementary; one is usually called the Watson strand, and the other is called the Crick strand. Primers were designed for amplifying the bisulfite-converted DNA sequences. The PCR primer information is listed in Table 3. PCR was performed in20-μl reactions, containing 5 ng of converted DNA, 5 μM of each primer, 4 mM dNTPs, 2 μl of 10xPCR buffer, and 1 unit of Taq DNA polymerase. Amplification was performed for 45 cycles using a GeneAmp PCR System 9600 (PE Applied Biosystems, Foster City, CA). PCR products were analyzed by 1% agarose gel electrophoresis to confirm successful amplifications.

### Analysis of DNA methylation

The purified DNA was subjected to DNA sequencing on the automated sequencer (ABI PRISM 3100 genetic analyzer) using Genescan 3.7 software (Applied Biosystems). Products were sequenced from both directions to validate each other. The methylation status at each CpG site was read out from a comparison between the sequences of the unconverted DNA, the converted Watson strand, and the converted Crick strand. The methylation percentage was quantitated from the Sanger sequencing results. Correlations between methylation patterns in different generations were analyzed by the spearman's rank correlation coefficient. The significance levels were set at 0.05 for all tests. The SAS statistical package 9.3 (SAS Institute, Inc., Cary, North Carolina) was used for all data managements and analyses.

## Author Contributions

Q.L. performed the experiments and wrote the first version of the manuscript. Y.C. and G.F. performed the statistical analysis. Y.M., J.R., W.L., W.X., L.M. and T.B. participated in the experiments. Y.W. and X.L. participated in study design. Q.S. and Q.L designed the work.

## Figures and Tables

**Table 1:** Spearman correlation analysis of the CpG methylation status between different generations of rats in the liver

*Strand*	*Variable*	*With Variable*	*Sample Correlation*	*Fisher's z*	*Bias Adjustment*	*Correlation Estimate*	*95% Confidence Limits*		*P-Value*
**Watson**	**Rat-1**	**Rat-2**	0.6775	0.8245	0.0154	0.6691	0.3547	0.8475	0.0002
	**Rat-1**	**Rat-3**	0.7337	0.9366	0.0167	0.7259	0.4476	0.8760	<.0001
	**Rat-1**	**Rat-4**	0.6768	0.8233	0.0154	0.6684	0.3536	0.8472	0.0002
	**Rat-1**	**Rat-5**	0.6217	0.7278	0.0141	0.6130	0.2687	0.8184	0.0011
	**Rat-2**	**Rat-3**	0.7464	0.9647	0.0170	0.7388	0.4695	0.8823	<.0001
	**Rat-2**	**Rat-4**	0.5029	0.5532	0.0114	0.4943	0.1031	0.7531	0.0134
	**Rat-2**	**Rat-5**	0.4664	0.5055	0.0106	0.4581	0.0566	0.7321	0.0238
	**Rat-3**	**Rat-4**	0.7975	1.0918	0.0181	0.7908	0.5618	0.9073	<.0001
	**Rat-3**	**Rat-5**	0.5979	0.6898	0.0136	0.5891	0.2336	0.8057	0.0020
	**Rat-4**	**Rat-5**	0.7028	0.8728	0.0160	0.6946	0.3957	0.8604	<.0001
**Crick**	**Rat-1**	**Rat-2**	0.5721	0.6506	0.0130	0.5633	0.1968	0.7917	0.0036
	**Rat-1**	**Rat-3**	0.5967	0.6881	0.0136	0.5879	0.2319	0.8050	0.0021
	**Rat-1**	**Rat-4**	0.7048	0.8768	0.0160	0.6967	0.3991	0.8615	<.0001
	**Rat-1**	**Rat-5**	0.7871	1.0638	0.0179	0.7802	0.5425	0.9023	<.0001
	**Rat-2**	**Rat-3**	0.7150	0.8974	0.0163	0.7070	0.4160	0.8666	<.0001
	**Rat-2**	**Rat-4**	0.6601	0.7929	0.0150	0.6515	0.3272	0.8385	0.0004
	**Rat-2**	**Rat-5**	0.6123	0.7126	0.0139	0.6035	0.2547	0.8134	0.0014
	**Rat-3**	**Rat-4**	0.8344	1.2025	0.0190	0.8286	0.6323	0.9249	<.0001
	**Rat-3**	**Rat-5**	0.5823	0.6660	0.0132	0.5735	0.2112	0.7972	0.0029
	**Rat-4**	**Rat-5**	0.7607	0.9979	0.0173	0.7533	0.4947	0.8894	<.0001

**Table 2:** Spearman correlation analysis of the CpG methylation status between different generations of rats in the kidney

*Strand*	*Variable*	*With Variable*	*Sample Correlation*	*Fisher's z*	*Bias Adjustment*	*Correlation Estimate*	*95% Confidence Limits*		*P-Value*
**Watson**	**Rat-1**	**Rat-2**	0.6817	0.8322	0.0155	0.6733	0.3614	0.8497	0.0002
	**Rat-1**	**Rat-3**	0.9392	1.7310	0.0213	0.9366	0.8542	0.9731	<.0001
	**Rat-1**	**Rat-4**	0.6626	0.7973	0.0151	0.6540	0.3311	0.8398	0.0004
	**Rat-1**	**Rat-5**	0.8148	1.1413	0.0185	0.8085	0.5944	0.9156	<.0001
	**Rat-2**	**Rat-3**	0.6339	0.7479	0.0144	0.6252	0.2869	0.8248	0.0008
	**Rat-2**	**Rat-4**	0.3253	0.3375	0.0074	0.3186	-0.1077	0.6460	0.1312
	**Rat-2**	**Rat-5**	0.5798	0.6622	0.0132	0.5710	0.2077	0.7959	0.0031
	**Rat-3**	**Rat-4**	0.6291	0.7399	0.0143	0.6204	0.2797	0.8223	0.0009
	**Rat-3**	**Rat-5**	0.8421	1.2284	0.0191	0.8365	0.6475	0.9285	<.0001
	**Rat-4**	**Rat-5**	0.6275	0.7372	0.0143	0.6188	0.2773	0.8214	0.0010
**Crick**	**Rat-1**	**Rat-2**	0.7046	0.8763	0.0160	0.6964	0.3986	0.8613	<.0001
	**Rat-1**	**Rat-3**	0.9526	1.8588	0.0217	0.9505	0.8851	0.9791	<.0001
	**Rat-1**	**Rat-4**	0.9437	1.7706	0.0215	0.9413	0.8645	0.9751	<.0001
	**Rat-1**	**Rat-5**	0.7705	1.0214	0.0175	0.7632	0.5122	0.8941	<.0001
	**Rat-2**	**Rat-3**	0.6324	0.7454	0.0144	0.6237	0.2847	0.8241	0.0009
	**Rat-2**	**Rat-4**	0.6887	0.8456	0.0157	0.6804	0.3728	0.8533	0.0002
	**Rat-2**	**Rat-5**	0.6385	0.7556	0.0145	0.6298	0.2939	0.8273	0.0007
	**Rat-3**	**Rat-4**	0.9457	1.7890	0.0215	0.9433	0.8691	0.9760	<.0001
	**Rat-3**	**Rat-5**	0.7784	1.0412	0.0177	0.7713	0.5265	0.8980	<.0001
	**Rat-4**	**Rat-5**	0.7695	1.0190	0.0175	0.7622	0.5104	0.8937	<.0001

**Table 3:** Primers

**Primers Name**	**For Bisulfite sequencing**	**For genomic sequence**
ashCRPtg96m1F	GGTTTTTGTATTATTGGTAGTAGG	GGCCCTTGTATCACTGGCAGCAGG
ashCRPtg366m1R	AAAAAACAAACTAACCCCTTCTC	GAGAAGGGGTCAGTCTGTTTCTC
ashCRPtg360m2F	AATTGGAGAAGGGGTTAGTTTG	AACTGGAGAAGGGGTCAGTCTG
ashCRPtg666m2R	ACTATATCCTATATCCTTAAACC	GGTCTAAGGATATAGGATACAGT
ashCRPtg665m3F	TGGTTTAAGGATATAGGATATAG	TGGTCTAAGGATATAGGATACAG
ashCRPtg895m3R	ACTAACTTCCTTCAAAATTCCC	GGGAACTTTGAAGGAAGCCAGT
ashCRPtg894m4F	TGGGAATTTTGAAGGAAGTTAG	TGGGAACTTTGAAGGCAGCCAG
ashCRPtg1087m4R	CTTCAAAACCCACAACTAAACC	GGCCCAGCTGTGGGTCCTGAAG
ashCRPtg1087m5F	GGTTTAGTTGTGGGTTTTGAAG	GGCCCAGCTGTGGGTCCTGAAG
ashCRPtg1321m5R	CAAAACACCTCAAATTCTAATTC	GAATCAGAATTTGAGGTGTTTTG
ashCRPtg1321m6F	GAATTAGAATTTGAGGTGTTTTG	GAATCAGAATTTGAGGTGTTTTG
ashCRPtg1557m6R	ACACCTAACCAATATCCTAATTC	GAATCAGGACACTGGCCAGGTGT
ashCRPtg1560m7F	TTAGGATATTGGTTAGGTGTTTG	TCAGGACACTGGCCAGGTGTCTG
ashCRPtg1779m7R	AACACTACAAACAAACAAAAACC	GGTTTTTGTTTGCTTGCAGTGCT
ashCRPtg1797m8F	TTGGTTTTTGTTTGTTTGTAGTG	CTGGTTTTTGTTTGCTTGCAGTG
ashCRPtg1950m8R	ATAAACTCCTCTAACAAAACACC	GGTGTCCTGTCAGAGGAGCCCAT
ashCRPtg1945m9F	ATAGGGTGTTTTGTTAGAGGAG	ACAGGGTGTCCTGTCAGAGGAG
ashCRPtg2359m9R	TCACAACTTCTAAAACAACTATC	GATAGTTGCTTTAGAAGTTGTGA
sshCRPtg25m1F	AATGGGAAGTGTAAATTTATAGGG	AATGGGAAGTGTAAACTTACAGGG
sshCRPtg446m1R	CATCTCCCCAACTCCCTATC	GATAGGGAGCTGGGGAGATG
sshCRPtg443m2F	TTTTAGATAGGGAGTTGGGGAG	CTCCAGATAGGGAGCTGGGGAG
sshCRPtg714m2R	TTACCCCAACAAAACAAATCTAC	GCAGATCTGCTCTGCTGGGGCAA
sshCRPtg714m3F	GTAGATTTGTTTTGTTGGGGTAA	GCAGATCTGCTCTGCTGGGGCAA
sshCRPtg895m3R	ACCCACATTCACAAAACTCTTC	GAAGAGCCCTGTGAATGTGGGC
sshCRPtg894m4F	TGAAGAGTTTTGTGAATGTGGG	TGAAGAGCCCTGTGAATGTGGG
sshCRPtg1199m4R	TACATTACAAACCTACTCCACC	GGTGGAGCAGGCCTGCAATGCA
sshCRPtg1206m5F	GTAGGTTTGTAATGTATATAGGG	GCAGGCCTGCAATGCATATAGGG
sshCRPtg1457m5R	CACCAAATAAAATTAACACCATC	GATGGTGTTAATCTCATCTGGTG
sshCRPtg1457m6F	GATGGTGTTAATTTTATTTGGTG	GATGGTGTTAATCTCATCTGGTG
sshCRPtg1701m6R	ATTCCTAAAATCACAATAACTCC	GGAGCTACTGTGACTTCAGGAAC
sshCRPtg1698m7F	ATTGGAGTTATTGTGATTTTAGG	ACTGGAGCTACTGTGACTTCAGG
sshCRPtg1991m7R	TCTAAAAAACCTCTCACATTTAC	GCAAATGTGAGAGGTTTCTCAGA
sshCRPtg1990m8F	AGTAAATGTGAGAGGTTTTTTAG	AGCAAATGTGAGAGGTTTCTCAG
sshCRPtg2239m8R	ACAAATAAAAACCACCCCAAAC	GCCTGGGGTGGCCCTTACCTGT
sshCRPtg2228m9F	ATTTTTTTTATAGTTTGGGGTGG	ATCTCTCCCATAGCCTGGGGTGG
sshCRPtg2394m9R	AAAAATCTAAAACTTCTAACCCC	GGGGCTAGAAGTCCTAGATCTCT
